# Congenital Diaphragmatic Hernia in a Case of Patau Syndrome: A Rare Association

**Published:** 2015-04-01

**Authors:** Jain A, Kumar P, Jindal A, Sarin Yk

**Affiliations:** 1 Department of Neonatology, Maulana Azad Medical College, New Delhi-110002; 2 Department of Pediatric Surgery, Maulana Azad Medical College, New Delhi-110002; 3 Department of Genetics, Unit of Pediatrics, Maulana Azad Medical College, New Delhi-110002

**Keywords:** Congenital diaphragmatic hernia, Patau syndrome, Congenital anomalies

## Abstract

Congenital diaphragmatic hernia (CDH) occurs in 5-10% associated with chromosomal abnormalities like, Pallister Killian syndrome, Trisomy 18, and certain deletions.. Association of CDH with trisomy 13 (Patau syndromes) is very rare. Here, we report such an unusual association, where surgical repair was done, but eventually the case succumbed as a result of multiple fatal co-morbidities.

## CASE REPORT

A 34 weeks male preterm weighing 1020 grams; an outcome of non-consanguineous marriage was delivered normally. He was born to a 35 years fourth gravida mother with one abortion in past, an old case of multi-drug resistant (MDR) tuberculosis. At birth, he was observed to have an aplasia cutis on scalp, wide fontanelle, bilateral post-axial polydactyly in hands, scaphoid abdomen and bilateral cryptorchidism. The face revealed low set ears, hazy corneas, and bilateral complete cleft lip with complete cleft palate (Fig. 1). The baby had right sided heart sounds with a basal systolic murmur. In view of respiratory distress and hypoxia he was shifted to Neonatal Intensive Care Unit (NICU) on oxygen. In view of progressive respiratory acidosis ventilator support was initiated. A requirement of maximum oxygen index (OI) of 12 was suggestive of favorable outcome. An echocardiography revealed a small (3 mm) atrial septal defect (ASD) and an inlet Ventricular Septal Defect (VSD). A left sided congenital diaphragmatic hernia (CDH) was confirmed on postnatal chest X ray.

**Figure:**
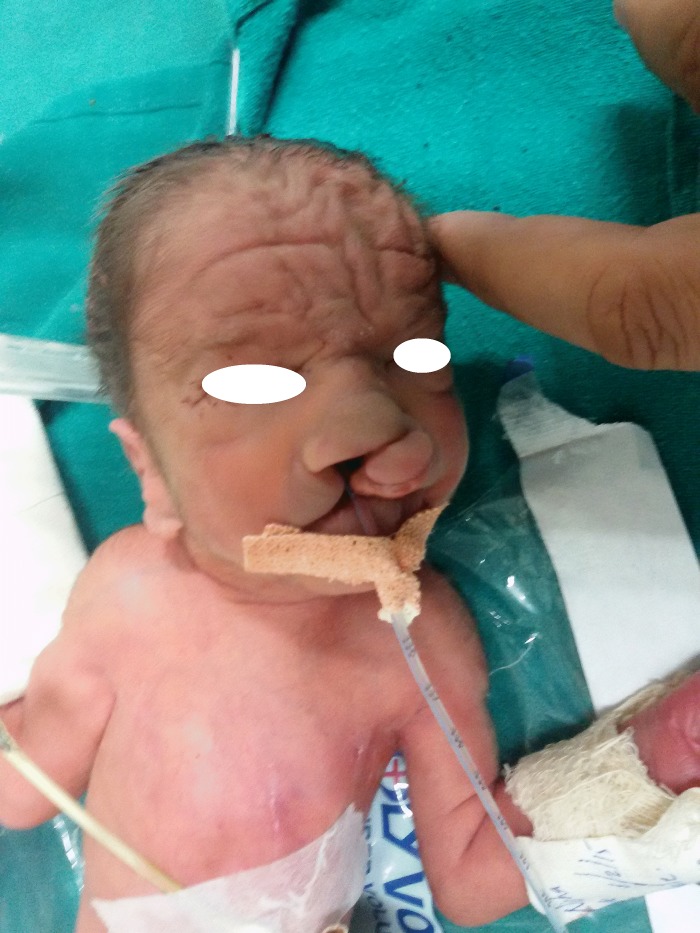
Figure 1: Bilateral cleft lip (Hare lip).

The surgery was done on the baby on the next day. Intraoperative findings suggested an isolated 2cm x 3cm defect in the left diaphragm with herniating spleen, splenic flexure of colon and a part of left liver. On day two, the baby developed shock with bleeding from multiple sites with thrombocytopenia which, promptly responded to inotropes and platelet transfusion and he was extubated to oxygen hood at 84 hrs. On fifth day, he had a refractory severe apnea requiring ventilator care. The workup for apnea was non suggestive, except for a mild asymmetric bilateral ventriculomegaly (Left more than right) with 2 small sub-ependymal cysts. He had stormy course in the NICU with progressive thrombocytopenia, acute kidney injury (AKI) and dyselectrolytemia. On eighth day of life, he had another refractory apnea followed by a fatal cardiac arrest. The karyotype report received after 3 weeks was suggestive of trisomy 13.

## DISCUSSION

Patau is a rare syndrome first recognized in 1960 [1]. It commonly results from, non- disjunction of chromosome 13 and rarely as a result of deletions, duplications and translocation [1]. Advanced maternal age is often seen as an association [2]. They have wide spectrums of anomalies ranging from mid-face to complex heart and brain malformations. The resuscitation is abandoned in these babies considering the associated lethal anomalies [3]. The median survival in spite of the aggressive management is less than 2 weeks and the karyotype report is seldom available [4]. 

Only 2 to 3 % of the trisomies survive to birth [4]. Our case even though had a history of spontaneous abortion; the genetic workup of fetus was unavailable. More than half of them have central nervous system, hearing, eyes, mouth, auricles, skin, hands, and feet, skeletal, cardiac, and genital abnormalities [5]. Diagnosis is often difficult, due to overlapping clinical findings with other syndromes notably trisomy 18 (Edwards). However, in our case the characteristic constellation of the findings suggested a possibility of Patau [6]. The newer modalities for early diagnosis like Fluorescent in Situ Hybridization (FISH), array-based comparative genomic hybridization (CGH) are costly and not widely available. We could only send for the conventional karyotype estimation. It is very difficult to decide and discuss the management plans of these babies with parents, without a confirmed diagnosis in hand. The syndromes like the anencephaly, trisomy 18 and 13, are suggested for “non-initiation of resuscitation” by most of the resuscitation guidelines considering their high mortality and universal morbidity [7]. Our case did not need any resuscitation at birth, even though he had features suggestive of Patau, but this was not confirmed at birth. Considering the willingness of the parents and the promising clinical course, we performed the repair of the CDH. With the antenatal anomaly scans and the biochemical screening it is now possible to confirm and diagnose these cases antenatally [8]. In such cases, where the diagnosis is confirmed at birth an alternate decision may be taken unlike our case. It is also advisable at this point to consider each case individually and offer appropriate diagnostics and genetic counseling to the parents.

## Footnotes

**Source of Support:** Nil

**Conflict of Interest:** Corresponding author is editor of the journal but he is not involved in decision making of the manuscript.

